# Multi-Finger Interaction and Synergies in Finger Flexion and Extension Force Production

**DOI:** 10.3389/fnhum.2017.00318

**Published:** 2017-06-19

**Authors:** Jaebum Park, Dayuan Xu

**Affiliations:** ^1^Department of Physical Education, Seoul National UniversitySeoul, South Korea; ^2^Institute of Sport Science, Seoul National UniversitySeoul, South Korea

**Keywords:** anticipatory synergy adjustment, multi-finger synergy, uncontrolled manifold hypothesis, finger flexion, finger extension

## Abstract

The aim of this study was to discover finger interaction indices during single-finger ramp tasks and multi-finger coordination during a steady state force production in two directions, flexion, and extension. Furthermore, the indices of anticipatory adjustment of elemental variables (i.e., finger forces) prior to a quick pulse force production were quantified. It is currently unknown whether the organization and anticipatory modulation of stability properties are affected by force directions and strengths of in multi-finger actions. We expected to observe a smaller finger independency and larger indices of multi-finger coordination during extension than during flexion due to both neural and peripheral differences between the finger flexion and extension actions. We also examined the indices of the anticipatory adjustment between different force direction conditions. The anticipatory adjustment could be a neural process, which may be affected by the properties of the muscles and by the direction of the motions. The maximal voluntary contraction (MVC) force was larger for flexion than for extension, which confirmed the fact that the strength of finger flexor muscles (e.g., flexor digitorum profundus) was larger than that of finger extensor (e.g., extensor digitorum). The analysis within the uncontrolled manifold (UCM) hypothesis was used to quantify the motor synergy of elemental variables by decomposing two sources of variances across repetitive trials, which identifies the variances in the uncontrolled manifold (*V*_UCM_) and that are orthogonal to the UCM (V_ORT_). The presence of motor synergy and its strength were quantified by the relative amount of *V*_*UCM*_ and V_ORT_. The strength of motor synergies at the steady state was larger in the extension condition, which suggests that the stability property (i.e., multi-finger synergies) may be a direction specific quantity. However, the results for the existence of anticipatory adjustment; however, no difference between the directional conditions suggests that feed-forward synergy adjustment (changes in the stability property) may be at least independent of the magnitude of the task-specific apparent performance variables and its direction (e.g., flexion and extension forces).

## Introduction

The design of the human hand enables us to execute a variety of dexterous hand actions through purposeful adjustment of finger motions or forces in various directions. A single muscle can generate and change its effect by pulling or relaxing along a straight line; however, complex and dexterous movements are generated by net actions of multi-muscles involved in a particular task (Bernstein, 1967; Turvey, 1990). Thus, hand dexterity is the ability to govern the net actions of multi-muscles involved in task, and the net actions of two groups of muscles create the overall finger flexion and extension by multi-fingers. It has been known that finger flexor muscles (e.g., extrinsic muscles including flexor digitorum profundus, superficialis, and intrinsic palmar muscles), which extend to the phalanges of fingers, develop relatively larger tension as compared to finger extensor (e.g., extensor digitorum, pollicis brevis, pollicis longus, etc) due to a larger cross-sectional area (CSA) in the flexors (Davies et al., 1988; Jacobson et al., 1992). Especially, the age-related effect on strength difference between finger flexion and extension was reported (Shim et al., 2007; Hsu, 2009; Kapur et al., 2010). The functions of the wrist flexor and extensor, which share their functions with the motions of the fingers, are partially specialized based on the fact that the physiological parameters (e.g., CSA, fiber length, etc.) of the muscles vary across flexors and extensors (Liu et al., 2014; Bertelli, 2015; Van Beek et al., in press). For human hands, individual fingers can be considered as separate but interdependent force actuators (Fahrer, 1981; Li et al., 2002; Lieu, 2008) for finger flexion and extension. “Interdependent force actuator” in fingers means that individual finger force generations are enslaved to some extent resulting in unintended force production (Zatsiorsky et al., 2000; Danion et al., 2003; Li et al., 2004; Martin et al., 2009). Because the interdependency and strength of individual fingers are varied between finger flexion and extension, the coordination patterns of individual finger forces may depend on the direction of finger force production.

In many of the daily hand and finger activities, we utilize two or more fingers together to achieve desired actions including net flexion or extension force generation. The use of multi-fingers implies that the individual fingers involved in tasks work together for the successful completion of the tasks. Therefore, the human hand with fingers is an excellent example of kinetic redundancy (Li et al., 1998a; Oliveira et al., 2006; Kim et al., 2008). The redundancy implies that the number of elements (fingers) is larger than the number of constraints; therefore, there are redundant degrees of freedoms (DOFs) in the description of the movement system (Bernstein, 1967; Latash, 2000). In multi-finger tasks, the number of constraints (i.e., the number of required conditions) given by experimental instruction (i.e., motor task) is typically smaller than the number of digits which were actively involved in the tasks (Li et al., 1998a; Zatsiorsky and Latash, 2008). Theoretically, therefore, an infinite number of force combinations of multi-fingers can equally be solutions for a specific performance such as total force or moment production. Recently, a computational approach to the coordinated behaviors of multi-elements has been proposed (Scholz and Schöner, 1999, 2014; De Freitas and Scholz, 2010). The main idea of this approach is that the controller may actively use a redundant set of elements resulting in solution families, and the idea is associated with the principle of motor abundance (Latash, 2000, 2012; Gera et al., 2010). It has been proposed that the organization of solution families for stable performance is a strategy used by a neural structure, which has been termed as “synergies” (Shim et al., 2003; Kang et al., 2004; Zhang et al., 2008). In general, the existence of synergic actions has been characterized and quantified by the task-specific co-variation across repetitive trials between redundant elements (Latash et al., 2002; Scholz et al., 2003; Friedman et al., 2009; Delis et al., 2013). Because finger flexor and extensor have different physiological properties, it is questionable whether the controller strategy that govern the multi-finger system in humans is contingent upon finger force directions.

Another aspect of synergic actions in the redundant human system is feed-forward modulation as a stability property. Stability in the human movement system refers to an ability to stabilize important performance variables in task-specific ways by organizing multi-elements in the system. Thus, “*good*” stability of the human movement system implies “*good*” ability for stabilizing the system against perturbations, which well fits with the classical definition of stability (Taga, 1995; Patla, 2004). In a redundant movement system, the stability of performance could be adjusted in both negative (i.e., destabilization) and positive ways without net mechanical outcomes. Indeed, a human being has an ability to adjust a certain neural-related variable(s) or to make a subtle change in a performance variable prior to a virtually detectable action if one knows in advance the information of “when” and “what” for the upcoming tasks (Aruin and Latash, 1995; Shiratori and Latash, 2001; Mohapatra and Aruin, 2013). Thus, the feed-forward adjustment is possibly implemented with proper information related to the timing and direction of the planned movements. The virtually detectable action is a consequence of mechanical effects (Kim et al., 2006; Monjo and Forestier, 2014) such as changes in force, torque, muscle activation (Li et al., 2002; Shim et al., 2006; Latash, 2010; Sarabon et al., 2013). Notably, if a performer is not aware of the timing of a future action, the feed-forward adjustment is not observed in a variety of human movements (Zhou et al., 2013; Togo and Imamizu, 2016), and this phenomenon has been termed as anticipatory synergy adjustments (ASAs). The phenomenon of feed-forward adjustment has been observed if the movement system is redundant, and the initiation of the change in the performance (i.e., mechanical effect) is triggered by a self-selected stimulation. Recent experiments have shown that the purpose of ASAs is to attenuate the strength of synergies prior to voluntary quick actions (i.e., rapid changes in net performance) (Krishnan et al., 2011; Kanekar and Aruin, 2014; Togo and Imamizu, 2016). It is assumed that the attenuation of the synergy is a purposeful destabilization of the performance in order not to compete for its synergy during a quick change in performances. Previous studies elucidated that the variations in the parameters of ASAs were associated with the strength of synergy and force production capabilities across various populations. For a healthy young group, a drop in the synergy index started about −200 ~−300 ms with respect to the initiation time for the apparent change in the performance (Olafsdottir et al., 2005; Shim et al., 2005; Kim et al., 2006; Klous et al., 2012). The activation time of ASAs is delayed with aging (Kapur et al., 2010; Wang et al., 2017), fatigue (Singh et al., 2010), and neurological disorders including Parkinson's diseases (Bleuse et al., 2008; Jacobs et al., 2009; Park et al., 2012, 2014; Jo et al., 2015), and cortical stroke (Sousa et al., 2015; Jo et al., 2016). Seemingly, the delayed ASAs were well-associated with the maximal voluntary contraction (MVC) and synergy strengths. However, parallel changes in the initiation time of the ASAs, synergy strength, and MVC were not obvious in stroke survivals (Jo et al., 2016) after strength training (Park et al., 2015), and in a comparison between men and women (Shim et al., 2004). We assumed that the movements of the finger flexion and extension are very different from their physiological and biomechanical perspectives. Therefore, it is worth investigating if the strength of synergy and its modulation are force direction and strength dependent measures by employing finger flexion and extension tasks.

In this study, we investigated both the flexion and extension efforts of finger actions and changed the force magnitude either to the same or opposite directions in a self-paced manner. We analyzed finger interdependency, multi-finger synergy, and modulation of the synergy index during finger flexion and extension. It is currently unknown whether the organization and feed-forward modulation of stability properties in multi-finger actions are affected by force directions and strengths. We hypothesized the following: (1) finger independency will be smaller during the finger extension effort rather than during the flexion effort. (2) the strength of the synergies for the steady-state force production will be stronger during the extension effort rather than during the flexion effort, and (3) the time of the ASAs will differ between conditions when the direction of force changes and when it does not change.

## Materials and methods

### Subjects

Nine right-handed young male subjects (height: 176.89 ± 6.74 m, mass: 77.89 ± 12.59 kg, age: 24.11 ± 3.69 years) were recruited in this study. The handedness of all participated subjects was right, which was determined by the Edinburgh Handedness Inventory (Oldfield, 1971). Prior to the experiment, we interviewed individual subjects to check their handedness and previous history of neuropathies or traumas to their upper extremities. None of the participants had a serious impairment history, and the study was performed in accordance with the recommendation of Seoul National University Institutional Review Board (IRB). The consent was informed, and all participants were requested to sign a consent form according to the procedure approved by Seoul National University IRB.

### Apparatus

**Figure 1:**
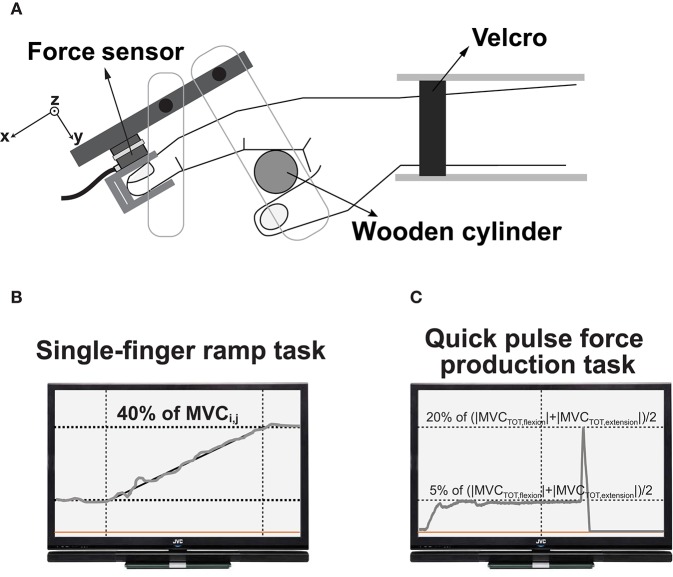
Top-down view of the experimental setup **(A)**. The subject's wrist was held stationary with Velcro straps. A wooden cylinder supported the palm, and the force sensors were attached to a frame. The feedback screen displayed the real-time finger forces and showed the templates during single-finger ramp tasks **(B)** and quick pulse force production tasks **(C)**.

The flexion and extension forces of four fingers along a single axis were measured with four force transducers (Model 208A03, PCB Piezotronics Inc., Depew, NY, USA) with amplifiers. The force transducers were mounted on a customized aluminum panel (size: 140 × 90 × 5 mm) which was fixed to a wooden board (Figure 1A). The panel positioned vertically to avoid the gravitational effect during finger force production. There were four straight slits anteroposteriorly on the aluminum panel to attach the force transducers. Adjacent slots were spaced mediolaterally by 3.0 cm along the *z*-axis. The subjects were supposed to insert their distal phalanges of the fingers into the thimbles (Figure 1A) such that it enabled the subjects to make isometric finger flexion or extension efforts selectively. The experimental frame including the panel and the sensors with the thimble was tilted at 25°. Thus, the initial posture of all the finger joints was slightly flexed. The force signals were conditioned, amplified, and an analog-to-digital converter was used to digitize the force signals at 500 Hz sampling rate using a customized LabView program (National Instrument, Austin, TX, USA).

### Procedures

The subject sat in a height adjustable chair facing computer monitor for a real-time force feedback. The subject abducted the upper arm by about 45° and flexed about 45° with the elbow flexion about 45°. The wrist position was positioned between pronation and supination with respect to the neutral position of the wrist. The total duration of the experiment for each subject was ~2 h. The 1-min break was given between every two trials.

There were three experimental blocks. The first block was the MVC tasks using single-finger and multi-fingers. Subjects were instructed to make isometric flexion or extension efforts as hard as possible with single-finger (*I, M, R, L*) and all four fingers (*TOT*), separately. During single-finger MVC tasks, the subjects were not allowed to lift non-task fingers on the corresponding sensors. The force feedback of the task-finger force was visualized on the computer screen (24-inches, 1,920 × 1,080 resolution at 60 Hz). Eight second were given to the subject to reach their maximum flexion or extension efforts. Each subject performed three trials at the MVC task for each condition. The maximal finger forces MVC_*i,j*;_*i* = {*I, M, R, L, TOT*}, *j* = {*flexion, extension*} were captured within 8 s, and MVC_TOT,*j*_ and MVC_*i,j*_ were used to decide target force levels in the next two tasks.

The second task, the single-finger ramp force production tasks, required the subject to make either extension or flexion effort (*j*) with one finger (*i*) and to follow the template shown in the screen. The template on the computer screen consisted of three phases including a 4-s horizontal line at 0% of MVC_*i,j*_, 12-*s* slanted line starting from 0 to 40% of MVC_*i,j*_, and the 4-s horizontal line at 40% of MVC_*i,j*_ (Figure 1B). The instruction to the subjects was that “keep all the fingers on the corresponding sensors and do not pay attention to unintended force production by non-task fingers.” We measured both task and non-task finger forces.

The third task was a multi-finger steady state (SS) force production followed by a quick pulse force production tasks. The subjects were given four combinations of “steady state” and “quick pulse” pairs regarding the directions of the finger forces: (1) flexion-flexion (FF), (2) flexion-extension (FE), (3) extension-flexion (EF), and (4) extension-extension (EE). The first letter in the naming of four conditions means the direction of finger force at SS, while the second letter means the direction of finger force for the quick pulse. Note that (2) and (3) conditions required to change the directions of finger force from flexion to extension or vice-versa in which they made a quick pulse force. The subjects were required to press the transducers with all four fingers simultaneously and to maintain a steady-state level for at least 5 s. After the SS force production, the subjects were instructed to produce a quick pulse force to the target within the next 5 s. Note that the initiation of a quick pulse force was not triggered by an external cue but by a self-selected cue. Also, the force direction information for both SS and quick pulse was given to the subject in advance using a template on the computer screen, so the subjects produced the tasks with in advance information of force direction and timing. The magnitudes of the SS force was set at 5% of (|MVC_TOT_,_flexion_|+|MVC_TOT_,_extension_|)/2. The change in force magnitude from “steady state” to “quick pulse” was set at 20% of (|MVC_TOT_,_flexion_|+|MVC_TOT_,_extension_|)/2 for all four conditions. In other words, the task space in the constrained flexion and extension force magnitudes was symmetrical, and magnitude difference between the constrained forces of SS and the quick pulse was the same for all four conditions. The computer screen for the real-time force feedback showed the first force level for the steady-state and the target force level for the quick pulse along the horizontal lines (Figure 1C). About 10–20 min practice time provided to each subject before data collection. The subject performed 25 for each condition. Thus, a total of 100 trials (25 trials × 4 conditions), and the 10-s break between every two trials was provided.

### Data analysis

#### Initial data processing

Customized MATLAB (MathWorks Inc., Natick, MA, USA) codes were written to process the measured force data. Before variable computation, the raw signals were digitally low-pass filtered at 10 Hz cut-off with zero-lag, 4th-order Butterworth filter. The following variables were computed to test the formulated hypotheses.

#### Enslaving matrix

The enslaving matrix (***E***) was calculated using the force data from the second task, the single finger force production tasks (Scholz et al., 2003; Kang et al., 2004). The elements in the ***E*** represent the relative amount of forces by non-task fingers, which assumed to be produced unintentionally, to the total force (F_TOT_) by all four fingers. A linear regression analysis was performed to compute the regression coefficients (*e* in Equation 1) between individual finger force (F_i_) and F_TOT_ over ramp duration. Then, 4 by 4 enslaving matrix (***E*** in Equation 2) was composed for flexion(*E*_flx_) and extension (*E*_ext_) task separately.

(1) $$\begin{array}{l} F_{i,j,k}=f_{i,k}^{0}+e_{i,j,k}\;F_{TOT,j,k} \end{array}$$

(2) $$\begin{array}{l} E_{k}=\left[\begin{array}{cccc} e_{I,I,k} & e_{I,M,k} & e_{I,R,k} & e_{I,L,k}\\ e_{M,I,k} & e_{M,M,k} & e_{M,R,k} & e_{M,L,k}\\ e_{R,I,k} & e_{R,M,k} & e_{R,R,k} & e_{R,L,k}\\ e_{L,I,k} & e_{L,M,k} & e_{L,R,k} & e_{L,L,k} \end{array}\right] \end{array}$$

where *i* = {I, M, R, L}, *j* = {I, M, R, L}, and *k* = {flx, ext}. *i* and *j* represent non-task and task finger, respectively. *k* indicates a force direction. *F*_*i,j,k*_ and *F*_*TOT,j,k*_ represent the individual *i*-finger force and total force by all four fingers, respectively, when *j*-finger was the task finger during *k*-force direction condition. Also, the averages of the off-diagonal components in both *E*_flx_ and *E*_ext_ for *j*-finger (task finger) were computed (*EN*_*i,k*_), which represent the total amount of finger force enslaving (Equation 3).

(3) $$\begin{array}{l} EN_{j,k}=\sum_{i}e_{i,j,k}/3(i\neq j) \end{array}$$

#### Quick pulse force production tasks

**Figure 2:**
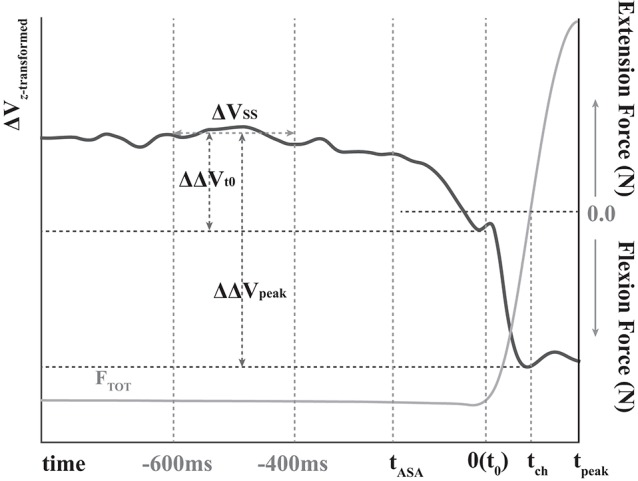
The sample data of total force (gray line) and variance of the total force (z-transformed ΔV, black line) during quick pulse force production tasks. Flexion forces are presented as negative, and extension forces are presented as positive. *t*_ASA_, *t*_0_, *t*_ch_, and *t*_peak_ stand for the time of anticipatory synergy adjustment (ASA), the time of initiation of total force(F_TOT_) change, the time of the direction of force changed, and of the peak pulse force, respectively. ΔV_SS_ represents average ΔV at a steady state. ΔΔV_t0_ and ΔΔV_peak_ stand for the change in the synergy index between steady state and *t*_0_ and between the steady state and negative peak of ΔV after *t*_0_, respectively.

Prior to the variable computation, we screened and deleted erratic trials to ensure that those trials did not sway the outcome variables. Especially, the trials, which showed multiple peaks or non-constant (not stabilized) force during steady state period, were excluded from the following analysis. The time of initiation of total force (*F*_*TOT*_) change (t_0_) was identified in the particular trial, and *t*_0_ was used as a reference time moment to calculate the time of the direction of total force (*F*_*TOT*_) changed (*t*_*ch*_) and of the peak pulse force (*t*_*peak*_) (Figure 2). *t*_0_ was quantified as the time when *dF*_*TOT*_/dt (the first derivative of the total force) reached 5% of its peak value in each trial. Further, average (AvgT) and standard deviation (SdT) of *t*_*ch*_ and *t*_*peak*_ across repetitive trials in each subject and condition were calculated. For the repetitive trials, the data were aligned with respect to *t*_0_, the time initiation of total force change. The co-variation of hypothetical commands to four fingers, i.e., multi-finger synergy, was quantified based on the uncontrolled manifold (UCM) hypothesis (Scholz and Schöner, 1999; Latash et al., 2002; Scholz et al., 2003, *for details see* Appendix in Supplementary Materials) using the sets of time aligned trials for each subject and condition. Finger modes (m), which are assumed to be the hypothetical commands to fingers, was computed using the ***E*** and individual finger forces for each time sample (Zatsiorsky et al., 2000). We assume that the hypothetical commands to four fingers are independent to each other and *E* matrix represent interdependency among finger force production. Thus, the independent commands (i.e., mode vector) to four finger can be achieved by multiplying two matrices, the inverse of *E* matrix and individual finger force vector (Equation 4). A mode vector reflects intended finger involvement of all four fingers by commands. Therefore, the UCM analysis with mode vectors enables us to infer how the neural commands to the fingers are organized to perform the given tasks (Scholz and Schöner, 1999; Latash et al., 2002). Since we assumed that the interdependency of finger force production was a direction-dependent quantity, we applied *E*_*flx*_ and *E*_ext_ selectively according to the direction of total finger force.

(4) $$\begin{array}{l} \text{m}={\left[\text{E}\right]}^{-1}F;F={\left[f_{I},f_{M},f_{R},f_{L}\right]}^{T} \end{array}$$

The variances of time series within two subspaces, *V*_*UCM*_ and *V*_*ORT*_, across the repetitive trials for each condition were quantified using force (*F*) and mode (m) vectors, separately. The mode or force vectors observed in the UCM space confirming to the variance (*V*_*UCM*_) across trials did not affect the magnitude of the performance variable. On the contrary, the orthogonal variance (*V*_*ORT*_) refers to the variability of performance variable across trials which was produced by the combination of finger forces. Briefly, a synergy index (Δ*V)* was quantified as the relative amount of *V*_*UCM*_ in the total variance, *V*_*TOT*_ (Equation 5). Note that the sum of *V*_*UCM*_ and *V*_*ORT*_ was equal to the total variance, *V*_*TOT*_. Further, the variances were normalized by the degrees of freedoms (DOFs) of the corresponding spaces (Latash et al., 2001; Friedman et al., 2009; Arpinar-Avsar et al., 2013).

(5) $$\begin{array}{l} \Delta V\left(t\right)=\frac{V_{UCM}\left(\text{t}\right)/3-V_{ORT}\left(t\right)/1}{V_{TOT}\left(t\right)/4} \end{array}$$

Before statistical tests, Fisher's *z*-transformation was applied to ΔVs (Δ*V*_*Z*_) since ΔVs were constrained by their computational boundaries. The steady state (SS) was set as the period 600–400 ms before *t*_0_ (Figure 2) (Park et al., 2013, 2014). Two indices were quantified in the period of ASA: the time of initiation of the ΔV drop (*t*_*ASA*_) and the change in the synergy index (ΔΔV_*t0*_) between SS and *t*_0_. Further, the drop magnitude of the synergy indices (ΔΔ*V*_*peak*_) between the SS and negative peak of ΔV after *t*_0_ (Figure 2) was quantified. The mean and standard deviation (*SD*) of Δ*V*_*Z*_ were computed over the period of SS; *t*_*ASA*_ was defined as the time when ΔV_Z_ dropped below its average SS value by more than 2SDs.

#### Statistics

Repeated-measured ANOVAs with the following factors: *Direction*_*SS*_ (force direction at the SS, two levels: flexion and extension), *Direction*_pulse_ (force direction at the quick pulse, two levels: flexion and extension), *Fingers* (four levels: index, middle, ring, and little fingers). The factors were selected for particular statistical tests. Mauchly's sphericity test was employed to confirm or reject the assumptions of sphericity. The Greenhouse-Geisser corrections were used when the sphericity assumption was rejected. The statistical power for all comparisons was computed, and for all planned comparisons, the power was over 0.7 from the pool of 9 subjects. The level of significance for all statistical tests was set at *p* < 0.05.

## Results

### Maximal voluntary contraction (MVC) force and finger independency

The MVC finger force during flexion was about two times larger than the MVC force during extension [*F*_(1, 8)_ = 23.35, *p* < 0.01]. On average (*n* = 9), the MVC_TOT_ forces during flexion and extension were 102 and 56 N, respectively. Individual finger forces were also larger during flexion than during extension. The order of individual finger MVC forces during flexion (MVC_*i,flexion*_) was I (40 N) > M (30 N), R (26 N) > L (17 N). The order of MVC_*i,extension*_ was I (22 N), M (19 N) > R (15 N), L (9 N). These findings were supported by a two-way repeated measure ANOVA with factors *Finger* (four levels: Index, Middle, Ring, and Little) and *Direction* (two levels: flexion and extension), which showed significant main effects of *Direction* [*F*_(1, 8)_ = 27.30, *p* < 0.01] and *Finger* [*F*_(3, 24)_ = 22.86, *p* < 0.01] with a significant interaction of *Finger* × *Direction* [*F*_(3, 24)_ = 3.86, *p* < 0.05].

**Figure 3:**
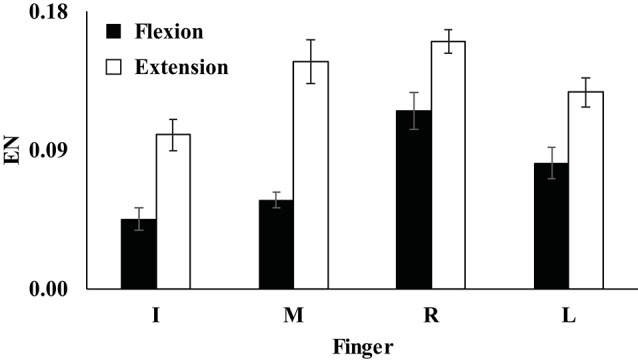
The index of enslaving (*EN*) of the index (I), middle (M), ring (R), and little (L) fingers during flexion (filled bars) and extension conditions (open bars). Average values are presented with standard error (SE) bars. *EN*_*I*_, *EN*_*M*_ < *EN*_*L*_ < *EN*_*R*_ during finger flexion (*p* < 0.05) and *EN*_*I*_ < *EN*_*M*_, *EN*_*R*_*, EN*_*L*_ during extension (*p* < 0.05).

Unintended finger force productions by non-task fingers during the single-finger ramp tasks were prominent during both finger flexion and extension. Further, the index of enslaving (*EN*) was computed, and the *EN* was larger in the extension condition than in the flexion condition for all four fingers (Figure 3). Also, *EN* of the index finger was smaller than the *EN*s of other three fingers for both flexion and extension conditions (Figure 3). A two-way repeated measure ANOVA with factors *Finger* (four levels: Index, Middle, Ring, and Little) and *Direction* (two levels: flexion and extension) showed main effect of *Direction* [*F*_(1, 8)_ = 73.44, *p* < 0.001] and *Finger* [*F*_(3, 24)_ = 16.36, *p* < 0.01] with a significant interaction of *Finger* × *Direction* [*F*_(3, 24)_ = 3.73, *p* < 0.05]. Pairwise comparisons showed that *EN*_*I*_, *EN*_*M*_ < *EN*_*L*_ < *EN*_*R*_ (*p* < 0.05) during finger flexion and *EN*_*I*_ < *EN*_*M*_, *EN*_*R*_*, EN*_*L*_ (*p* < 0.05) during extension (Figure 3), which reflected a significant *Finger* × *Direction*.

### Timing indices

**Figure 4:**
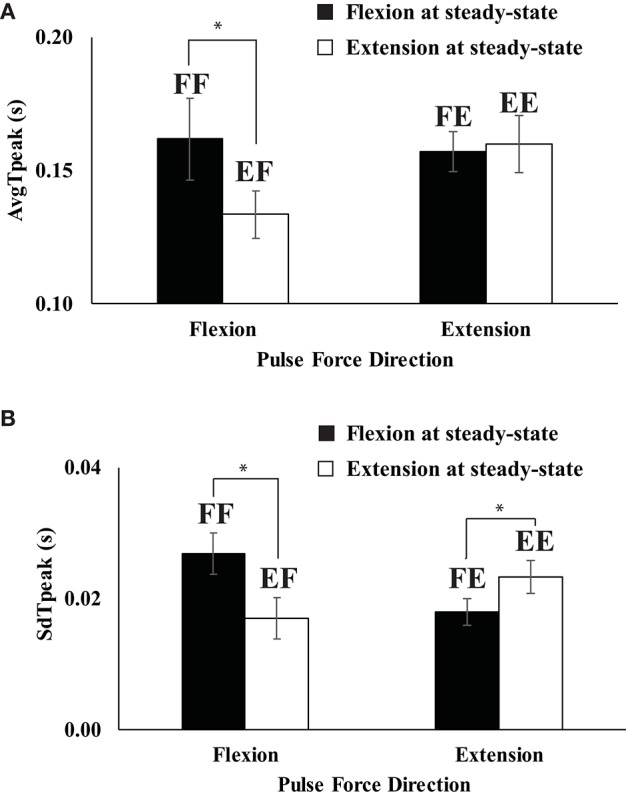
Average (AvgT_peak_) **(A)** and standard deviation (SdT_peak_) **(B)** of the time to reach peak pulse force across repetitive trials when the direction of steady state force was flexion (filled bars) and extension (open bars). Two capital letters above the bars represent the experimental conditions. The first letter represents the force direction at the steady state, and the second letter stands for the direction of quick pulse force. “F” and “E” stand for flexion and extension, respectively (e.g., FF represents “flexion” to “flexion”). Averaged across subjects data are shown with standard error bars. The asterisks (^*^) show statistically significant differences between conditions (*p* < 0.05).

An average time to reach peak pulse force (AvgT_peak_) across repetitive trials was faster in *EF* (extension to flexion) than in *FF* (flexion to flexion) (Figure 4A) while there was no difference on AvgT_peak_ between *FE* (flexion to extension) and *EE* (extension to extension). AvgT_peak_ for *EF* and *FF* were 0.135 and 0.162 s, respectively. The standard deviation of *t*_*peak*_ (SdT_peak_) across repetitive trials was smaller when the conditions required to change the direction of finger force from flexion to extension or vice-versa for a quick pulse force production. In other words, SdT_peak_ of *EF* (extension to flexion) was smaller than that of *FE* (flexion to extension), and SdT_peak_ of *FE* (flexion to extension) < SdT_peak_ of *EE* (extension to extension) as shown in Figure 4B. These findings were supported by a two-way repeated measure ANOVA with factors *Direction*_SS_ (two levels: flexion and extension) and *Driection*_pulse_ (two levels: flexion and extension), which showed the main effect of *Direction*_SS_; *F*_(1, 8)_ = 6.74, *p* < 0.05] on AvgT_peak_ with a significant *Driection*_SS_ × *Driection*_pulse_ [*F*_(1, 8)_ = 6.74, *p* < 0.05]. Two main effects on SdT_peak_ were not significant, while the factor interaction *Direction*_SS_ × *Driection*_pulse_ on SdT_peak_ was significant [*F*_(1, 8)_ = 4.21, *p* < 0.05]. The significant interaction reflected the fact that SdT_peak_ of *FF* > SdT_peak_ of *EF* (*p* < 0.05) and SdT_peak_ of *FE* < SdT_peak_ of *EE* (*p* < 0.05). In addition, we computed the average (AvgT_ch_) and standard deviation (SdT_ch_) of time of change in the direction of force (*t*_*ch*_) with respect to *t*_0_, the time of initiation of *F*_*TOT*_ change. AvgT_ch_ was 0.098 for the *EF* condition (extension to flexion) and 0.109 for the *FE* condition (flexion to extension), but the difference did not reach statistical significance [*F*_(1, 8)_ = 2.83, *p* = 0.13]. There was no significant effect on SdT_ch_.

### Multi-finger synergy indices in mode space

**Figure 5:**
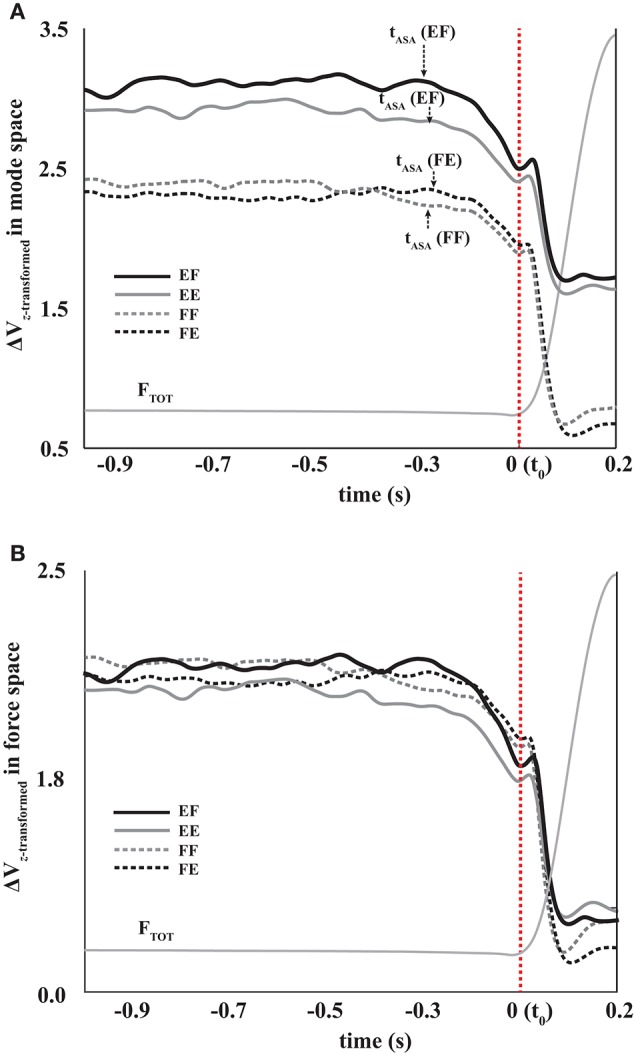
The total force (F_TOT_, thin gray lines) and *z*-transformed synergy index (ΔV_Z_) during FF (gray dotted line, flexion to flexion), FE (black-dotted line, flexion to extension), EF (black solid line, extension to flexion), and EE (gray solid line, extension to extension) in mode space **(A)** and force space **(B)**. Averages across subjects are presented for ΔV_Z_. The times of ASA initiation (t_ASA_) are shown with the *arrows*.

**Figure 6:**
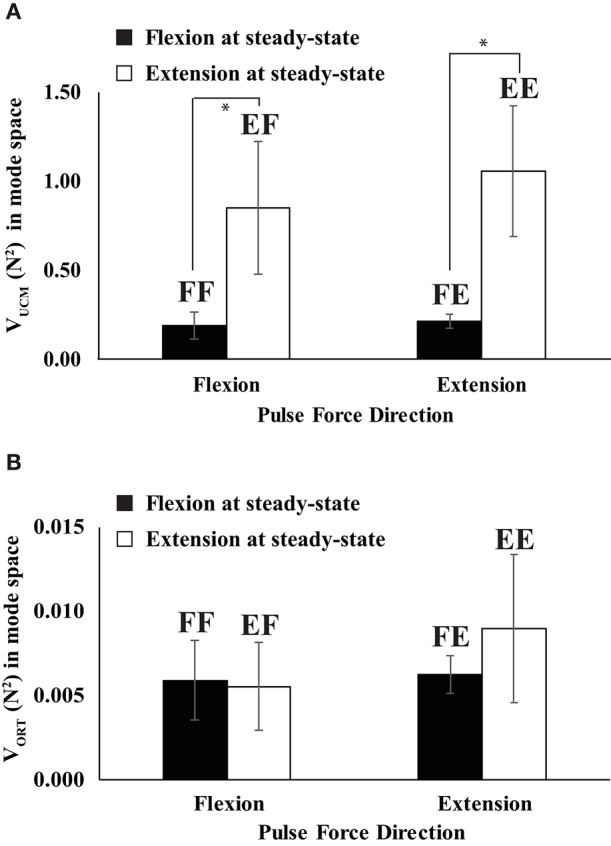
The variances in the UCM (*V*_*UCM*_) **(A)** and orthogonal space (V_ORT_) **(B)** when the direction of steady state force was flexion (filled bars) and extension (open bars). Two capital letters above the bars represent the experimental conditions. The first letter represents the force direction at the steady state, and the second letter stands for the direction of quick pulse force. “F” and “E” stand for flexion and extension, respectively (e.g., FF represents “flexion” to “flexion”). Averages across subjects data are shown with standard error (SE) bars. The asterisks (^*^) show statistically significant differences between conditions (*p* < 0.05).

In the mode space, the indices of the steady state (SS) force stabilization synergies (ΔV_SS_) were larger during the extension effort than during the flexion effort regardless of the directions of peak pulse force (i.e., *EF* & *EE* > *FF* & *FE* in Figure 5A), which was confirmed by the main effect of *Direction*_SS_ [*F*_(1, 8)_ = 26.77, *p* < 0.01] without a significant interaction of *Direction*_SS_ × *Direction*_pulse_. In general, the variance in the UCM (*V*_*UCM*_) is larger than the variance in the orthogonal space (V_ORT_) for all four conditions at the SS (*p* < 0.01), which confirmed the existence of the force stabilizing synergy (Figure 6). Thus, ΔV_SS_ difference between the flexion and extension conditions in the mode space was mainly caused by the larger *V*_*UCM*_ during the extension effort than the flexion effort, and there was no significant difference in V_ORT_ between the conditions. This result was confirmed by two-way repeated-measure ANOVAs separately on *V*_*UCM*_ and V_ORT_ with factors *Direction*_SS_ (two levels: flexion and extension) and *Direction*_pulse_ (two levels: flexion and extension). The main effect of *Direction*_SS_ on *V*_*UCM*_ was significant without a factor interaction [*F*_(1, 8)_ = 5.68, *p* < 0.05] (Figure 6A), while main effects and factor interaction were not statistically significant on V_ORT_ (Figure 6B).

**Figure 7:**
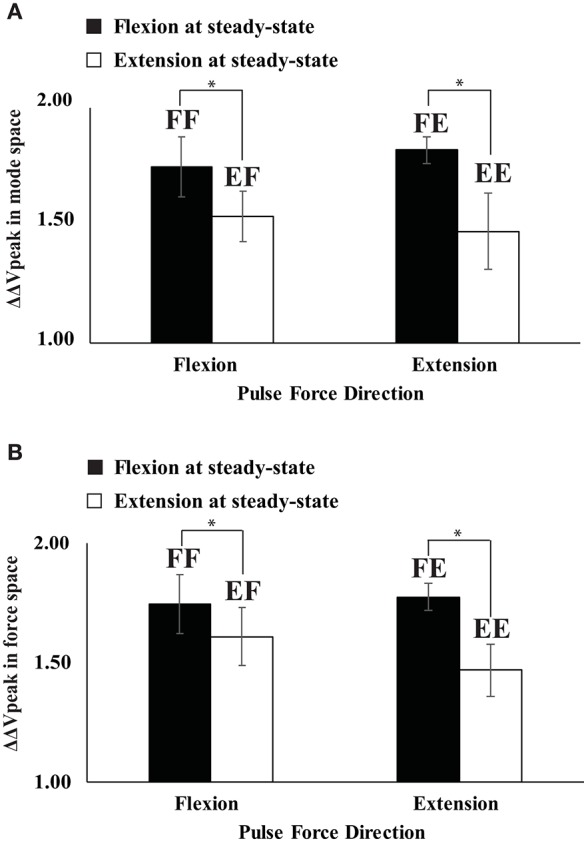
The difference in the synergy index between steady state and negative peak (ΔΔ*V*_peak_) for four conditions of FF (flexion to flexion), FE (flexion to extension), EF (extension to flexion), and EE (extension to extension) in mode space **(A)** and force space **(B)**. Averaged data across subjects with standard error (SE) bars are presented. The asterisks (^*^) show statistically significant differences between conditions (*p* < 0.05).

In all four conditions, it was evident that the synergy index started to drop before the initiation of the force pulse and reached its negative peak before the time of F_peak_. We quantified three indices during and after the anticipatory synergy adjustment, the time of the anticipatory synergy adjustment (t_*ASA*_), the difference in the synergy indices between SS and *t*_0_ (ΔΔ*V*_*t*0_), and the difference in the synergy indices between SS and negative peak (ΔΔ*V*_*peak*_). There was no significant difference on *t*_ASA_ (Figure 5A) and ΔΔ*V*_t0_ between the conditions with no factor interactions. On average, *t*_ASA_ was about 0.28 s, and the magnitude of the drop in the synergy index during the ASA was not statistically different between the conditions. The ΔΔ*V*_*peak*_ was larger when the direction of finger force at the SS was a flexion (i.e., a larger drop in the flexion), and this result was not affected by the direction of finger force at the quick pulse. A two-way repeated measure ANOVA with factors *Direction*_SS_ (two levels: flexion and extension) and *Driection*_pulse_ (two levels: flexion and extension) supported these finding confiming the main effect of *Direction*_SS_ [*F*_(1, 8)_ = 13.60, *p* < 0.05] on ΔΔ*V*_*peak*_ without a significant interaction (Figure 7A).

### Multi-finger synergy indices in force space

The same set of variables in the mode space analysis was used for the analysis using the data in the force space. In general, ΔV, *V*_*UCM*_, V_ORT_ at the SS, and ΔΔV_t0_ were not statistically different between the conditions, and factor interactions were also not significant (Figure 5B). However, ΔΔ*V*_*peak*_ was larger when the direction of finger force at the SS was a flexion, which was similar to ΔΔ*V*_*peak*_ pattern in the mode space analysis (Figure 7B). This finding was supported by a two-way repeated measure ANOVA with factors *Direction*_SS_ (two levels: flexion and extension) and *Driection*_pulse_ (two levels: flexion and extension), which showed the main effect of *Direction*_SS_ [*F*_(1, 8)_ = 10.17, *p* < 0.05] on ΔΔ*V*_*peak*_ without a significant interaction.

## Discussion

The two hypotheses formulated in the Introduction were supported by the results of this study. The third hypothesis about the anticipatory synergy adjustment during flexion and extension was rejected. The actions of fingers were more independent (i.e., smaller enslaving index, *EN*) during finger flexion than during finger extension, which supports the first hypothesis. The second hypothesis was supported by the results that the strength of synergy index was larger for extension than for flexion in the mode space although the force space analysis showed no statistical difference. The anticipatory synergy adjustment was observed in all four experimental conditions although there was no significant difference between the conditions, which rejects the third hypothesis. The following sections will focus on the possible mechanism of the force direction-dependent changes in the finger independency, stability indices, and anticipatory changes in the stability properties.

### Finger independency during finger flexion and extension

Unlike the actions of robotic fingers where individual fingers have separate actuators, the fingers of a human hand cannot make actions independently (Häger-Ross and Schieber, 2000; Zatsiorsky et al., 2000; Lang and Schieber, 2004) resulting in “voluntary” but “unintended” finger actions. This observation has been termed as enslaving or enslavement (Li et al., 1998b, 2004). The enslaving has been used as an index of finger interdependency and attributed to biomechanical and central factors. The biomechanical factors include an anatomical connection (i.e., passive connection) within the hand and forearm, which induces mechanically coupled actions of fingers (Häger-Ross and Schieber, 2000; Schieber and Santello, 2004). The central factors for the enslaving include divergence and convergence of cortical projections due to overlapping digit representation in the hand area of the primary motor cortex (Schieber, 1990; Schieber and Hibbard, 1993). A series of previous studies has reported a significant relationship between the voluntary force production capability (i.e., MVCs) and finger force enslaving. One group of studies reported a positive correlation between enslaving and MVC, which is likely enslaving increased by the magnitude of the MVC (Danion et al., 2000, 2001; Shinohara et al., 2003), which was opposite to the results of this study. The subjects in those studies were healthy individuals with different levels of finger strengths (e.g., young, healthy-aged elderly, females, and fatigued subjects). The other group of studies provided evidence of the counterexamples of a positive correlation between MVC and enslaving. A higher enslaving was observed in individuals with neurological disorders such as Parkinson's disease, cerebellar disorder, and stroke (Cho et al., 2010; Park et al., 2013; Jo et al., 2016) whose MVC forces were smaller than age- and gender-matched controls. The higher enslaving with lower MVC forces in groups with neurological diseases is probably led by central factors rather than peripheral (biomechanical) factors (Park et al., 2013, 2014). However, it is still questionable as to the relative contributions of peripheral and central factors to enslaving (and MVC forces) in healthy peoples. In our study with healthy individuals, the higher enslaving was accompanied by a lower MVC force during finger extension, which was a similar enslaving pattern to that found in patients with neurological disorders. By combining the abovementioned studies, the higher enslaving during finger extension may be a consequence of both peripheral and central reasons; however, the contribution of the supraspinal mechanism to finger individuation may be relatively small. The peripheral reason for the relatively smaller enslaving during finger flexion may include the extensor mechanism, which produces an extension action at the distal interphalangeal joint due to a structure of passive connective tissues (Li et al., 2001). Furthermore, it has been reported that the forces in the action of the extensor mechanism reduce the intrinsic hand muscles and bone contact force at the metacarpophalangeal joint (MCP) (Hu et al., 2014), which may give rise to a positive effect on independent movements of individual fingers. One of the central reasons may be a consequence of a greater corticospinal projection ratio to the finger flexor muscles (Chye et al., 2010).

### Multi-finger synergies in force and mode spaces

As we have already discussed, a higher enslaving during extension implies that fingers act less independently, which is accompanied by a relatively larger unintended force production. Furthermore, by comparison with the synergy indices (ΔV) in the force and mode space analyses, the synergy indices for the force stabilization during the steady state force production was larger during finger extension than during finger flexion, while the two space analyses were not statistically different (see Results Multi-finger synergy indices in mode space and Multi-finger synergy indices in force space). These results suggested that finger forces were coupled relatively stronger during finger extension by the abovementioned factors (i.e., peripheral and central), while hypothetical commands (i.e., finger mode) to the fingers showed a stronger negative covariation (i.e., larger positive ΔV) during finger extension compared to finger flexion. Indeed, corticomotor excitability was affected by the directional constraints of movements (McMillan et al., 2006) and was relatively larger for the extensor muscles than for the flexor muscle (Palmer and Ashby, 1992). As such the findings suggested that corticomotor excitation is assumed to be purposeful actions for the planned movements, and could combine with the fact that a stronger co-variation between neural commands during finger extension in this study. The interdependency among finger actions leads to positive correlations between finger forces even though commands to finger (i.e., mode) are independent. The stabilization of the performance variable (i.e., total force) is generally achieved by negative co-variation between individual finger forces. In other words, the negative co-variation between elements (e.g., forces or modes) is a typical strategy to compensate performance errors elicited by elements resulting in stable net force production. Thus, a strong interdependency of finger actions contributes to the positive relationship among finger actions, which increases the demand for a proper co-variation (i.e., error compensation) among the fingers for a stable total force production.

In addition, there were two distinctive characteristics of the time profile of the net forces and stability indices during finger flexion and extension. First, the time to produce a quick pulse force was faster and consistent when the pulse force direction was opposite to the direction of the steady-state force (*EF* & *FE* conditions. see Result). Note that gravity is not an issue when interpreting the result because the experimental frame in this study was vertically oriented so that the finger flexion and extension forces were gravity-free measures. It is well-known that electromechanical delay (i.e., EMD, the delay between an electrical state in the muscles and a mechanical action within the human body; Corcos et al., 1992) is shorter during eccentric muscle contraction (Cavanagh and Komi, 1979). Eccentric contraction can be observed where a counter-movement occurs prior to a primary movement (Komi and Bosco, 1978). The shorter and consistent force production in the *FE* and *EF* conditions could be associated with shorter electromechanical delay (EMD). The other distinctive observation is that the decreased in the magnitude of stability (ΔΔV_peak_) indices during a quick change in total force was larger in the conditions of *FF* (flexion to flexion) and *FE* (flexion to extension) compared to the conditions for *EE* (extension to extension) and *EF* (extension to flexion) in both the force and mode space analyses. In other words, the level of destabilization for a quick change in the overall performance may depend on the direction of the force before the rapid change and not the force direction intended for the quick change. It seems that the decreased in the magnitudes of the stability indices rely on the history of the finger force direction for stable force production and not on the direction of future actions. Thus, the immediate history-dependent changes of stability indices may be associated with neuromuscular hysteresis (Partridge, 1965; Joyce et al., 1969; Gielen and Houk, 1984).

### Anticipatory synergy adjustment

The human control system, the central nervous system (CNS), is capable of changing the stability properties of the performance in advance or capable of changing the performance directly against a predictable perturbation (Kim et al., 2006; Shim et al., 2006; Olafsdottir et al., 2008; Wang et al., 2017). Notably, the predictable perturbation should induce a mechanical effect resulting in changes in the salient performance variables. In the human movement system, there are two different, but complementary, types of feed-forward movement control, and those are anticipatory synergy adjustment (ASA) and anticipatory postural adjustment (APA). The main observation when implementing ASA is the destabilization of the system (i.e., a decrease in the synergy index) against a predictable change in a salient performance variable, which has been well-documented by previous studies (Klous et al., 2011; Sousa et al., 2015; Piscitelli et al., 2016). In a redundant system, the destabilization, which could be achieved by the change in the co-variation pattern of the elements, could result in close to zero net mechanical effect due to extra degrees of freedom (DOF) with respect to the DOFs of the tasks. Therefore, ASA as a feed-forward adjustment used by the CNS adjusts the synergy parameters (i.e., stability properties) without obvious changes in the net performance. In contrast, APA emphasizes changes in the performance variables such as muscle activations and the net force/moment prior to the upcoming perturbation. Previous experiments have shown that APAs were observed in average muscle activation patterns in a time series and that APA induces changes in the salient performance variables (Latash et al., 1995; Shiratori and Latash, 2001; Sousa et al., 2015; Piscitelli et al., 2016). In particular, APA is observed after the occurrence of ASA in regard to its timing. The function and timing of APA and ASA are different, but it has been assumed that a single or similar neural mechanism has a role in both phenomena.

Earlier studies reported parallel changes in the synergy indices and ASA with various populations and treatments including aged-group (Kapur et al., 2010; Wang et al., 2017), Parkinson's disease patients (Bleuse et al., 2008; Jacobs et al., 2009; Park et al., 2012, 2014; Jo et al., 2015), patients with the cerebellar disorder (Park et al., 2013), and effect of vibration on the intrinsic hand muscles (Arpinar-Avsar et al., 2013). Another group of studies have reported non-parallel changes in synergy indices and ASAs. Cortical stroke survival showed the delayed ASA but no difference in the strength of synergy as compared to the control subjects (Cho et al., 2013). Additionally, the synergy indices and muscular strength increased with wrist strength-training (Park et al., 2015) while there was no difference in ASAs after the training (Olafsdottir et al., 2008). These two groups of studies have suggested that the subcortical structure including the cortico-basal-thalamocortical circuit has a critical role in both the formation and adjustment of the stability properties for the successful completion of tasks. Furthermore, it seems that the neural mechanism of ASAs may not be affected by the strength of the peripheries, which was also shown by no difference in ASAs between men vs. women (Shim et al., 2004). The results of this study suggest that the mechanism of ASAs may be strength- and direction-independent quantity and the neural process of feed-forward adjustment could be a separate process controlling the co-variation between the elements.

## Author contributions

All of the authors contributed in a fair and equal manner throughout the process of experiment, analysis, and manuscript writing. JP designed and executed the experiment, and JP and DX took the significant share of the further experiment and data analysis. JP and DX all participated in the writing of the first draft.

### Conflict of interest statement

The authors declare that the research was conducted in the absence of any commercial or financial relationships that could be construed as a potential conflict of interest. The reviewer KK and handling Editor declared their shared affiliation, and the handling Editor states that the process nevertheless met the standards of a fair and objective review
