# Supplementary material for: Multi-Finger Interaction and Synergies in Finger Flexion and Extension Force Production
Source: Front Hum Neurosci. 2017 Jun 19;11:318. doi: 10.3389/fnhum.2017.00318 (PMC5474495; doi:10.3389/fnhum.2017.00318)
Supplement: Supplementary file 1 [file DataSheet1.docx]

# Appendix

*Uncontrolled manifold (UCM) analysis (see Latash et al. 2002; Scholz et al. 2002 for details)*

The UCM analysis was performed for performance variable ($F_{TOT}$) with four individual finger forces at mode and force space.

For $F_{TOT}$, changes in the forces of individual fingers sum up to produce a change in $F_{TOT}$:

$dF_{TOT}=\left[ \begin{matrix} 1 & 1 & 1 & 1 \end{matrix} \right]*\boldsymbol{E}\cdot{[\begin{matrix} dF^{I} & dF^{M} & dF^{R} & dF^{L} \end{matrix}]}^{T}$ (1)

The UCM was defined as an orthogonal set of the vectors e_i_ in the space of the individual finger forces that did not change the averaged $F_{TOT}$across trials:

$0=\left[ \begin{matrix} 1 & 1 & 1 & 1 \end{matrix} \right]*\boldsymbol{E}\cdot e_{i}$ (2)

These directions were found by computing the null-space of the Jacobian of this transformation ($\left[ \begin{matrix} 1 & 1 & 1 & 1 \end{matrix} \right]*\boldsymbol{E}\cdot e_{i}).$ For the force space analysis, an interdependency between finger forces was not excluded, therefore, the Jacobian of the transformation was ($\left[ \begin{matrix} 1 & 1 & 1 & 1 \end{matrix} \right]\cdot e_{i})$.

$e_{i}$is the basis vector spanning the linearized UCM (the null space of the Jacobian). The mean-free forces were then projected onto these directions and summed to produce:

$f_{||}=\sum_{i}^{n-p} (e_{i}^{T}\cdot df)\cdot e_{i}$ (3)

where ‘*n* = 4’, which corresponds to the number of degrees-of-freedom of the elemental variables, and ‘*p* = 1’, which is the number of degrees-of-freedom of the performance variable $(F_{TOT})$. The component of the de-meaned forces orthogonal to the null-space is given by:

$f_{\perp}=df-f_{||}$ (4)

The amount of variance parallel to the UCM space is computed by:

$V_{UCM}=\frac{\sum{|f_{||}|}^{2}}{N_{trials}}$ (5)

The amount of variance orthogonal to the UCM is:

$V_{ORT}=\frac{\sum{|f_{\perp}|}^{2}}{N_{trials}}$ (6)

The normalized difference between these variances is quantified by a variable ΔV:

$\Delta V=\frac{(\frac{V_{UCM}}{n-p}-\frac{V_{ORT}}{p})}{(\frac{V_{TOT}}{n})}$ (7)

where V_TOT_ stands for the total variance, and the variances are further normalized by the number of degress of freedom in corresponding spaces.
